# Clinical Relevance of Serum Vascular Endothelial Growth Factor and Interleukin-6 in Patients with Colorectal Cancer

**DOI:** 10.4103/1319-3767.80378

**Published:** 2011

**Authors:** Ayman Eldesoky, Ashraf Shouma, Yousef Mosaad, Amira Elhawary

**Affiliations:** Department of Internal Medicine, Specialized Medical Hospital, Faculty of Medicine, Mansoura University, Egypt; 1 Department of General Surgery, Mansoura Faculty of Medicine, Mansoura University, Egypt; 2 Department of Clinical Pathology, Clinical Immunology Unit, Faculty of Medicine, Mansoura University, Egypt; 3 Department of Pathology, Mansoura University Hospitals, Faculty of Medicine, Mansoura University, Egypt

**Keywords:** Clinical relevance, colorectal cancer, interleukin-6, vascular endothelial growth factor

## Abstract

**Background/Aim:**

Some biological factors play a role in stimulation of malignant growth, metastasis and angiogenesis; however, their clinical relevance has not yet been well established for most of them. This work was aimed at studying the clinical relevance of serum vascular endothelial growth factor (VEGF) and interleukin -6 (IL-6), in patients with colorectal cancer (CRC).

**Materials and Methods:**

Preoperative serum levels of VEGF and IL-6 were measured by enzyme -linked immuno -assay in 35 CRC patients and in 30 healthy controls.

**Results:**

CRC patients with or without metastasis had significantly higher VEGF and IL-6 levels than healthy controls (all *P* < 0.001). Patients with advanced clinical stage had significantly higher levels of VEGF and IL-6 than those with early clinical stage (all *P* < 0.001). Also, patients with metastatic disease had significantly higher VEGF and IL-6 levels than those with localized disease (all *P* < 0.001). The diagnostic accuracy for invasiveness was 83% for VEGF (cut off value = 240 pg/ml) and 66% for IL-6 (cut off value = 6.7 pg/ml), with sensitivity 79% and 74% and specificity 68% and 59%, respectively.

**Conclusion:**

In CRC patients, preoperative measurement of serum VEGF and Il-6 may prove useful non-invasive diagnostic indicators associated with advanced clinical stage and tumor metastasis that warrants further investigations.

Angiogenesis, the formation of new blood vessels from endothelial precursors, is a prerequisite for the development, growth, and progression of solid malignancies.[1] Vascular endothelial growth factor (VEGF) is one of the most potent endothelial cell mitogens that plays a critical role in angiogenesis. Detection of circulating VEGF has been investigated as a potential serum diagnostic marker for malignant disease and for inflammation.[2] Increased serum concentrations of free VEGF have been measured in various types of cancers.[3]

Recently, it is becoming clear that the large amount of cytokines and growth factors released during inflammation by immune and non-immune cells may influence the carcinogenesis process. Interleukin-6 (IL-6) and interleukin -23, cytokines which play key roles in the induction and maintenance of gut inflammation during inflammatory bowel diseases, have been shown to influence the development and growth of colitis associated colorectal cancer (CRC).[4] IL-6 is an immunomodulatory cytokine,[5] which also plays a role in growth stimulation, metastasis, and angiogenesis in secondary tumors in a variety of malignancies,[6] including CRC.[7–11]

CRC is the second leading cause of cancer -related deaths in the USA and Europe.[12] Many attempts have been made to define the biological profile of CRC in order to improve early diagnosis and the prognostic stratification, and eventually find a cure.[13,14]

## Aim

This work was designed to study the clinical relevance of serum VEGF and IL-6 in patients with CRC.

## MATERIALS AND METHODS

### Patients

Between August 2006 and June 2009, 35 CRC patients were selected from the attendants of the outpatient’s clinics and endoscopy unit of the Specialized Medical Hospital and from referrals of the Oncology Center, Mansoura University. The included patients comprised 26 males and 9 females with an average of age 49 ± 13.2 years. Thirty healthy subjects (23 males and 7 females) were selected on voluntary basis and included as the control group. Patients with history or clinical evidence of chronic infections, immunological disorders, recent pregnancy, trauma, surgery (within 1 month), or any other malignances were excluded.

The study protocol conforms to the Medical Sciences Ethics Committee of Mansoura Faculty of Medicine and all the included patients and selected controls had their written informed consent. Medical history included in addition to the demographic data, symptoms suggestive of colonic neoplasm (abdominal pain, bloody stools, fever, anemia and loss of weight), history of colonic troubles and family history of CRC. Clinical examination included both general and local abdominal examination with per rectal examination. A series of investigations were performed including laboratory tests (complete blood count, ESR, liver function tests, serum creatinine, fasting blood glucose, and carcinoemberyonic antigen) and radiological assessments (abdominal ultrasound, barium enema, and abdominal computed tomography).

Elective colonoscopy was performed following routine preparation with recoding of the observed colonic lesions and obtaining mucosal biopsies from the tumor tissues and the adjacent normal mucosa for histopathological assessment. Tumors were graded according to the pathological features (TNM classification).[15]

### Sample collection

Preoperative serum samples were obtained from the included 35 CRC patients once the diagnosis was established. Serum was also collected from 30 healthy volunteers using the same procedure as for the cancer patients. The control group comprised 7 females and 23 males with an age range of 59 ± 10.9 years. To avoid pre -analytical sample-to-sample variation due to blood collecting procedures, each blood sample was allowed to clot for at least 4 h before collecting the serum, which was immediately frozen and stored at -80 °C until used for the measurement of VEGF and IL-6 levels.[16]

### Quantification

For quantitative measurements of serum VEGF and IL-6 levels, we used the Quantikine quantitative human VEGF and IL-6 sandwich enzyme -linked immunosorbent assay kits (R and D system, Abington, UK for VEGF and Minneapolis, Minnesota for IL-6), which are specific for VEGF and IL-6, respectively. All the analysis and calibrations were performed in duplicate according to manufacturer’s instructions. The calibration on each microtiter plate included recombinant human VEGF and IL-6 standards. Optical density was measured using a microtiter plate reader at 450 nm. The blank was subtracted from duplicate readings for each standard and sample. The VEGF and IL-6 concentration was reported in pg/ml.

### Statistical analysis

Statistical analysis was done using the SPSS 10.0 software package. The comparison of continuous variables in two different subgroups was performed by the using non -parametric Mann-Whitney *U*-test. Comparison of the continuous variables in three different subgroups was performed using the ANOVA test. The diagnostic performance of both VEGF and IL-6 was assessed by receiver operating characteristic curve. *P* ≤ 0.05 was considered statistically significant.

## RESULTS

The results of patient’s characteristics showed that CRC patients with metastasis had significantly higher percentage of abdominal discomfort, ESR (*P* = 0.02, and 0.005, respectively) than CRC patients with localized lesions [Table 1]. Patients with advanced clinical stages had significantly higher levels of VEGF and IL-6 than those with early clinical stage (*P* < 0.001) [Table 2].

**Table 1:** Characteristics of the studied patients with colorectal cancer

	Total (n = 35)	Localized (n = 12)	Metastatic (n = 23)	*P* value
History				
Diarrhea	13 (37)	3 (25)	10 (43)	0.28
Blood in stool	31 (89)	9 (75)	22 (96)	0.06
Abdominal discomfort	28 (80)	7 (58)	21 (91)	0.02
Significant weight loss	17 (49)	5 (42)	12 (52)	0.55
Smoking	33 (94)	12 (100)	21 (91)	0.29
Positive family history	8 (23)	3 (25)	5 (22)	0.82
Laboratory				
HB (g/dl)	10.3±0.9	10.8±1.6	9.9±2.1	0.2
ESR (1st hour)	41±16	34±7	46±13	0.005
Colonoscopy				
Proximal colon	13 (37)	5 (42)	8 (35)	0.68
Distal colon	20 (57)	7 (58)	13 (57)	0.91
Multiple	2 (6)	0	2 (9)	0.29

Figures in parentheses are in percentage

**Table 2:** Comparison of serum VEGF and IL-6 between colorectal cancer patients and controls

		No.	Median VEGF		Median IL-6	
			pg/ml	*P*	pg/ml	*P*
Control	Total	30	-	0.21	-	0.52
	Male	23	82±13.3		2.9±0.8	
	Female	7	75±10.5		2.7±0.3	
Colorectal cancer	Total	35		0.86	-	0.9
	Male	26	299±176		7.58±2.7	
	Female	9	287±195		7.46±2.1	
Clinical stage	T1	16	213.4±32.2	<0.001	5.17±2.1	<0.001
	T2-T4	19	434.2±76.7		8.23±2.6	

*Mann-Whitney *U*- test (*P* value), VEGF: vascular endothelial growth factor, IL-6: interleukin-6

CRC patients with or without metastasis had significantly higher VEGF and IL-6 levels than controls (all *P* ≤ 0.001). Patients with metastatic disease had significantly higher VEGF and IL-6 levels than those with localized disease (*P*< 0.001) [Table 3].

**Table 3:** Comparison of serum VEGF and IL-6 levels between the studied groups

Group	VEGF (pg/ml)	*P* value	IL-6 (pg/ml)	*P* value
Control (n =30)	78.1 ± 12.6	P1 < 0.001	2.96 ± 0.7	P1 < 0.001
Localized(n = 12)	108.7±17.3	P2 < 0.001	4.45±1.9	P2 < 0.001
Metastasis(n = 23)	518.6±138.5	P3 < 0.001	8.8±2.4	P3 < 0.001

*P*1: Localized group versus control group; *P*2: Group with metastasis versus control group; *P*3: Group with metastasis versus localized group, VEGF: vascular endothelial growth factor, IL-6: interleukin-6

Using cutoff values 240 pg/ml for VEGF and 6.7 pg/ml for IL-6, the diagnostic accuracy for invasiveness was found to be 83% and 66% respectively, with sensitivity 79% and 74% and specificity 68% and 59%, respectively [Table 4].

**Table 4:** Diagnostic accuracy, sensitivity, and specificity of serum VEGF and IL-6 in colorectal cancer patients

	VEGF	IL-6
Cut off value (pg/ml)	240	6.7
Diagnostic accuracy (%)	83	66
Sensitivity (%)	79	68
Specificity (%)	74	59

ROC curve with 95% CI

## DISCUSSION

CRC patients had higher VEGF levels than healthy controls. Also, significantly higher serum VEGF was found in CRC patients with advanced clinical stages than in those with an early clinical stage, and in patients with metastasis than those with localized cancer. These findings run in parallel with previous reports where, the metastatic colon cancer expressed significantly more VEGF gene than localized tumors.[8,17] The occasional discrepancies in the results of the different studies could be explained by certain factors, e.g. inflammation, tumor infiltrating cells, and number of platelets in the serum sample.[18] Patients with high serum VEGF had high pathological tumor stage and regional lymph node metastasis. VEGF could modulate lymph vessel density and microvessel density that correlate with the malignant potential of tumors, patient survival and could be a useful tool for the selection of postoperative management and treatment strategies in patients with CRC.[19] Therefore, high serum VEGF could be considered as an usher for advanced disease state and tumor metastasis in CRC patients. Similar to the findings of Ueda *et al*., who reported that an increase in the serum level of VEGF was shown to be a predictor of metastasis in gastrointestinal tumors.[20]

There is a dynamic relationship among tumor cells, inflammatory cells, cytokines, and chemokines.[21] Cytokines and chemokines within tumors can contribute to the progression of tumors to a more aggressive metastatic phenotype or play a role in tumor therapy.[17,22] IL-6 is most often classified as a proinflammatory cytokine, although IL-6 and IL-6 -regulated acute phase proteins were previously suggested as anti-inflammatory and immunosuppressive, and may negatively regulate the acute -phase response.^23^

Our results proved higher levels of IL-6 in patients with CRC than healthy controls. Similarly, higher levels of IL-6 were found in metastatic than non -metastatic CRC patients. Our results are in agreement with the previous studies that reported increased circulating levels of IL-6 in patients with advanced pathological stages of CRC.[10] Previous correlation between tumor tissue expression of IL-6 and serum concentration in CRC implies that increased systemic IL-6 might be a result of local tumor production.[24] These findings have encouraged us to suggest the possible role of IL-6 as a trigger for the disseminated cells to develop into metastatic tumors. We ask for more clinical trials addressing the investigation of the novel genetic and pharmacologic agents including anti -angiogenic agents giving hope for future therapies which could be suitable for advanced CRC patients.

IL-6 can be released from tumor infiltrating leukocytes,[25] but is produced to a large extent by tumor cells themselves. In human colon cancer, IL-6 expression parallels tumor progression, reaching a maximum in high grade cancerous lesions.[9] In addition, IL-6 increases invasiveness of colon cancer cells and likely promotes secondary tumor formation through its angiogenic potency. The role of IL-6 in promoting progression and metastatic spread of colon cancer depends not only on the extent of basal but also, importantly, on the extent of inducible IL-6 expression at certain stages of tumor development.[10] Thus, IL-6 could serve as autocrine and paracrine growth factor for CRC, and high serum level of this cytokine might correlate with the poor prognosis and the increased production of angiogenic factors.

IL-6 has been shown to correlate with platelet count and platelet VEGF content. As circulating VEGF is mostly transported by platelet, IL-6 could be regarded as an indirect angiogenic factor that facilitates the production and distribution of VEGF to the metastatic sites. The importance of IL-6 on VEGF metabolism has been confirmed.[4] Both VEGF and IL-6 possibly have a role in stimulation of CRC growth and metastasis. An association between VEGF and IL-6 has been previously reported in carcinomas of the breast.[26] Although the biological mechanisms linking metastatic CRC with the increase in the serum levels of IL-6 and VEGF are not clearly understood, the findings of this study support a possible link between the advanced clinical disease and these cytokines.

In conclusion, in the CRC patients, raised serum VEGF and IL-6 may prove valuable non-invasive diagnostic indicators associated with advanced clinical stage and tumor metastasis that warrants further investigations.
